# The Influence of Alkoxy Substitutions on the Properties of Diketopyrrolopyrrole-Phenyl Copolymers for Solar Cells

**DOI:** 10.3390/ma6073022

**Published:** 2013-07-22

**Authors:** Zandra George, Renee Kroon, Robert Gehlhaar, Gabin Gbabode, Angelica Lundin, Stefan Hellström, Christian Müller, Yves Geerts, Paul Heremans, Mats R. Andersson

**Affiliations:** 1 Department of Chemical and Biological Engineering/Polymer Technology, Chalmers University of Technology, SE-412 96 Gothenburg, Sweden; E-Mails: zandra@chalmers.se (Z.G.); renee.kroon@chalmers.se (R.K.); angelica@chalmers.se (A.L.); stefan.hellstrom@borealisgroup.com (S.H.); christian.muller@chalmers.se (C.M.); 2 Organic Electronics, PV/PMEPV/OPV, IMEC vzw, Kapeldreef 75, B-3001 Leuven, Belgium; E-Mails: gehlhaar@imec.be (R.G.); heremans@imec.be (P.H.); 3 Université Libre de Bruxelles, Laboratoire de Chimie des Polymères, CP 206/1, Boulevard du Triomphe, B-1050 Brussels, Belgium; E-Mails: gabin.gbabode@univ-rouen.fr (G.G.); ygeerts@ulb.ac.be (Y.G.)

**Keywords:** organic solar cells, conjugated polymers, DPP, synthesis, Density Functional Theory (DFT)

## Abstract

A previously reported diketopyrrolopyrrole (DPP)-phenyl copolymer is modified by adding methoxy or octyloxy side chains on the phenyl spacer. The influence of these alkoxy substitutions on the physical, opto-electronic properties, and photovoltaic performance were investigated. It was found that the altered physical properties correlated with an increase in chain flexibility. Well-defined oligomers were synthesized to verify the observed structure-property relationship. Surprisingly, methoxy substitution on the benzene spacer resulted in higher melting and crystallization temperatures in the synthesized oligomers. This trend is not observed in the polymers, where the improved interactions are most likely counteracted by the larger conformational possibilities in the polymer chain upon alkoxy substitution. The best photovoltaic performance was obtained for the parent polymer: fullerene blends whereas the modifications on the other two polymers result in reduced open-circuit voltage and varying current densities under similar processing conditions. The current densities could be related to different polymer: fullerene blend morphologies. These results show that supposed small structural alterations such as methoxy substitution already significantly altered the physical properties of the parent polymer and also that oligomers and polymers respond divergent to structural alterations made on a parent structure.

## 1. Introduction

Polymer based organic photovoltaics have attracted a lot of attention as potential renewable energy technology in the last decades [1,2,3,4]. Low cost and fast roll-to-roll production, in combination with light-weight and flexible devices are advantages that make polymer solar cells interesting and a potential competitor to traditional silicon-based devices. So far, the most successful polymer solar cells are bulk heterojunction-type devices which employ a mixture of an electron donating polymer and an electron withdrawing fullerene as the active layer. The performance of the devices has increased rapidly in the last few years with power conversion efficiencies now at 8%–10% [5,6,7,8,9].

In the last few years, diketopyrrolopyrrole (DPP)-based polymers have emerged as a promising material for both thin-film transistors and solar cells, reaching power conversion efficiencies of around 5% [10,11,12,13,14]. The synthesis of DPP can be performed in a few simple steps from commercial products, making it an attractive material for photovoltaic devices. Since DPP is a planar unit it promotes π–π stacking, thereby potentially resulting in high charge carrier mobility [15]. The π–π stacking and optical properties of the material can be tuned by adding different donor units to the DPP-copolymer backbone [16,17]. Attachment of alkyl side chains to the nitrogen atoms in the DPP unit improves the solubility of the material, which is important for solution process ability and the film forming ability of the polymers [18].

Bijleveld *et al*. recently reported a copolymer based on 2,5-bis(2-hexyldecyl)-3,6-di(thiophen-2-yl)pyrrolo[3,4-c]pyrrole-1,4(2H,5H)-dione and benzene, which displayed a band gap of 1.55 eV. Photovoltaic devices based on a blend of the so-called PDPPTPT (hereafter P1) and PC_71_BM reached good power conversion efficiency of 5.5% after optimization with a processing agent [19]. If this polymer is modified with alkoxy side chains on the phenyl spacer a redshifted absorption will be obtained. The alkoxy side chains would also result in a higher molecular weight while the oxygen would shift the highest occupied molecular orbital (HOMO) level towards vacuum and reduce the energy gap [20,21,22,23].

However, it is well established that structure-property relationships are usually not straightforward as a structural alteration usually alters additional properties aside from the desired ones. Each of these additionally changed properties could have an impact on the final performance of a device. For instance, longer side chains are commonly employed to improve the solubility and molecular weight but also influence solid state aggregation of the polymer and the resulting blend morphology when employed with fullerenes in solar cells [24,25,26]. Employing longer side chains potentially results in decreased device performance through the insulating effect of the side chains, which can hinder the movement of charges, as well as a good donor–acceptor contact [27]. Various groups have reported on the influence of various polymer properties, such as molecular weight and solvent quality, on optical absorption [28,29].

Therefore, it is important to investigate not only if alterations on the polymer structure induce the desired change in optical or electronic properties, and relate specifically these to performance, but, in addition, that the same assessment is done for the additionally altered properties. In an attempt to ascertain structure-property relationships more specifically we synthesized P1 derivatives with short methoxy (P2) and long octyloxy (P3) side groups on the benzene ring, as well as well-defined oligomers based on P1 and P2. This allowed us to attribute several altered physical and optical properties to either the alkoxy substitution or conformational effects in the polymer.

## 2. Results and Discussion

### 2.1. Synthesis and Physical Properties of Oligomers and Polymers

**Scheme 1:**
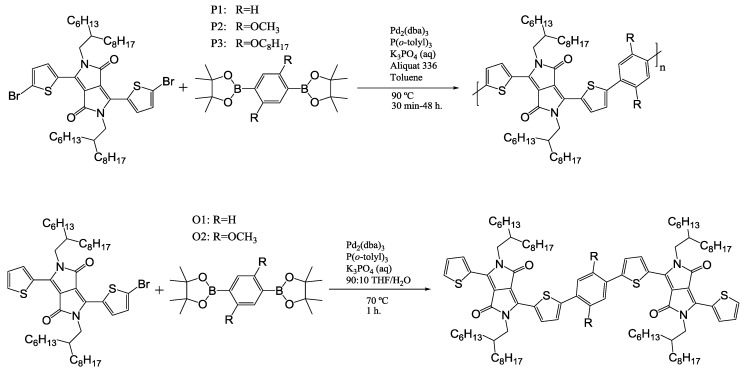
Chemical structure and synthesis of polymers and oligomers.

All polymers and oligomers were synthesized via Suzuki polymerization (Scheme 1), which resulted in rather reasonable molecular weights. All materials were analyzed by thermogravimetric analysis (TGA) and differential scanning calorimetry (DSC) (Table 1). P3 exhibits a much higher molecular weight since addition of octyloxy side chains improves solubility. TGA (Supplementary Information Figure S1) indicates that all polymers are quite thermostable, but also that the introduction of alkoxy side groups lowers the thermal stability somewhat.

**Table 1:** Physical properties of oligomers and polymers.

Material	M_n_ (kg/mole) ^a^	PDI	TGA (°C) ^b^	T_m_ (°C)	T_c_ (°C)
			*1% wt. loss*		
P1	15	1.5	415	>350	>350
P2	12	2.7	336	>300	260
P3	29	2.3	347	240	190
O1	–	–	257	153	110
O2	–	–	341	181	148

^a^ Measured against polystyrene standard in TCB at 135 °C; ^b^ under nitrogen atmosphere.

DSC (Supplementary Information Figure S1) shows only very weak and rather broad glass transition temperatures which prevents attributing a value to it. An endothermic transition on heating and exothermic transition upon cooling is detected for P2 and P3, which is indicative for transferring between a more disordered (heating) or more ordered (cooling) state in the material. The endothermic transition of P2 could not be observed in the DSC thermogram, which is attributed to degradation (T_d, 1%_ = 336 °C) perturbing the measurement. The evolution of the endothermic (and exothermic) transition for this series of polymers, T_c_: P1 > P2 > P3, is expected since most polymers exhibit reductions of such transition temperatures upon alkyl substitution due to increased flexibility of the polymer chain, whether this is originating from the backbone or the side chains [30]. As a side note, since the T_m_ of P3 is reduced to well below the degradation temperature, melt-processing would be another option to explore in the future.

O1 and O2 (Scheme 1) were synthesized and analyzed by DSC to investigate the effect of the different phenyl spacer on the observed trend in the polymers’ thermal transitions while excluding molecular length as a factor. T_g_ for both oligomers could again not be determined by DSC. For both oligomers T_m_ and T_c_ were observed, which is 28 °C higher for O2 and opposite of the trend observed for the polymers (Supplementary Information Figure S2). The origin of this surprising result was investigated via density functional theory (DFT)-calculations, described in the next section. This trend in thermal behavioris most likely not continued in the polymers P1 and P2 due to reasons of symmetry, where rotation of one phenyl relative to the next in the chain results in the same chain conformation while rotation of the dimethoxy spacer offers different chain conformations [31], thereby hindering the ability of a material to order.

DFT (B3LYP/6-31G (d,p)) calculations shows that O2 generally is slightly more planar than O1, see Table S1. Several configurations of the O1 and O2 were investigated, which show up to 0.098 eV difference for O1 and 0.116 eV for O2. The most stable configuration of O1 is when the sulphur on the thiophene is trans towards the carbonyl group on the DPP unit, see Scheme S1. For the O2 oligomer the most stable configuration is also when the sulphur on the thiophene and the methoxygroup on the benzene spacer is cis, see Scheme S1. This configuration, with the sulphur on the thiophene and the methoxy group on the benzene spacer is generally ~10 degrees more planar than the opposite configuration, see Table S1. The rotational barriers for the O1 and O2 oligomer are between 0.17 and 0.44 eV. This trend, with more planar conformers of O2, would give rise to higher ordering and thus higher endothermic and exothermic transitions at higher temperatures. In addition, a larger dipole moment introduced by the methoxy group could also offer an additional driving force to facilitate the ordering of the material.

X-ray diffraction (XRD, Supplementary Information Figure S3) on polymer powders shows that all polymers exhibit some lamellar ordering, P1 > P2 > P3, which seems to correlate with the alkoxy side chain length.

### 2.2. Electrochemical and Optical Properties

The influence of alkoxy substitution on the energy levels of the polymers was investigated by square wave voltammetry (SWV, Supplementary Information Figure S4). As a result of the electron donating nature of the alkoxy substitution, the oxidation potential of P2 and P3 was shifted towards a vacuum. However, even though P2 and P3 exhibit similar optical absorption, there still exists a large difference between the oxidation potential of P2 and P3. We attribute this difference to the fact that electrochemistry is sensitive to many factors (e.g., varying ion transport through the polymer film due to the thickness) and provides only an estimate. Therefore, we additionally calculated the HOMO levels by subtracting the optical energy gap from the LUMO energy (Table 2). The combined SWV/UV-Vis results indicate that the HOMO is shifted towards vacuum by approximately 0.2 eV, predicting a lower V_oc_ for P2 and P3 compared to P1 since the HOMO_polymer_–LUMO_acceptor_ difference influences the energy of the CT-state [32].

**Table 2:** Optical and electrochemical data.

Polymer	λ_max_ (nm)	λ_onset_ (nm)	E_g, onset_ (eV)	HOMO ^a^ (eV)	LUMO (eV)
P1	751	816	1.52	−5.10	−3.58
P2	718	925	1.34	−4.89	−3.55
P3	773	943	1.32	−4.88	−3.56

^a^ calculated via lowest unoccupied molecular orbital (LUMO) −E_g, opt_.

**Figure 1:**
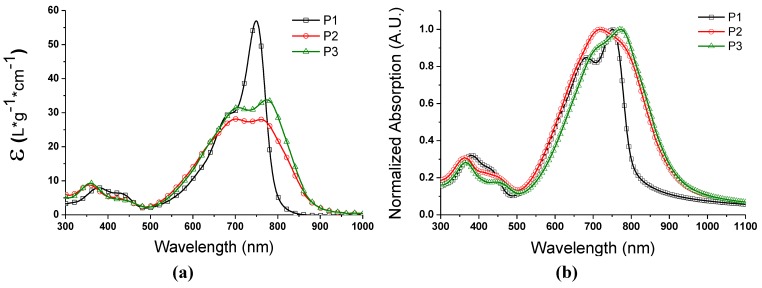
UV-Vis absorption of (**a**) dilute polymer solutions (CHCl_3_, ~16 mg/L); and (**b**) solid state, spun from ~10 mg/mL CHCl_3_ solutions.

The introduction of electron-donating alkoxygroups results in a broader and 100 nm redshifted absorption onset for P2 and P3, both in chloroform solution (Figure 1a) and thin film (Figure 1b). The low energy absorption consists of two main contributing peaks, a low energy blueshifted peak and a low energy redshifted peak. In an attempt to compare the absorptivity of the polymers, the solution absorption coefficient in chloroform was determined, described by Beaujuge *et al.* [33]. In our case, an additional correction for the diluting effect due to the additional alkyl side chains in P2 and P3 was done. We note that, even though comparing the absorption is done for a homologous series of polymers, this method only offers an estimate due to uncertainties in the effective conjugation length. The solution absorption measurements indicate that P1 has a higher absorption coefficient at the absorption maximum compared to both alkoxy substituted polymers, which show broader but less strong absorption. This could be attributed to a stiffer backbone of P1, which either promotes intrachain aggregation or a more rod-like behavior, which decreases the conformational distribution in the polymer chain and improves the effective conjugation length.

UV-Vis spectra of O1 and O2 CHCl_3_ solutions and thin films show a redshifted absorption but no absorption broadening (Supplementary Information Figure S5). This leads us to believe that the origin of the absorption broadening for the polymers is indeed from increased conformational distribution in the alkoxy-substituted polymers.

To obtain a featureless solution absorption profile we dissolved the polymers in chloronaphthalene (CN, Supplementary Information Figure S6), a solvent known for its good solubilizing properties regarding conjugated polymers. Hot polymer: CN solutions seem to result in solutions by showing a blueshift in absorption and the disappearance of the dual peak absorption feature upon cooling, and the same absorption profile obtained from chloroform solution was obtained. Alkoxy substituted materials continued to display a redshift compared to P1. In addition, CN seems to be a worse solvent compared to chloroform, at least for the P1 polymer, since large aggregates appear when cooling the solution to RT (Supplementary Information Figure S7) while the chloroform solutions do not show any visible aggregation.

The difference in redshift and maximum peak positions between dilute solution and thin film absorption is rather small for all polymers, which indicates that the source of these transitions has to be quite similar in both environments. On going from solution to solid state, the blueshifted absorption contribution seems to increase more with decreasing solubility. This can be explained by the use of a fast drying solvent such as chloroform combined with higher polymer concentrations and reduced solubility when producing polymer thin films. “Freezing” the chains faster into an amorphous state would then increase the amorphous contribution relative to a more extended/ordered chain conformation, thus the blue shifted absorption contribution would increase.

### 2.3. Device Performance and Atomic Force Microscope (AFM)

**Figure 2:**
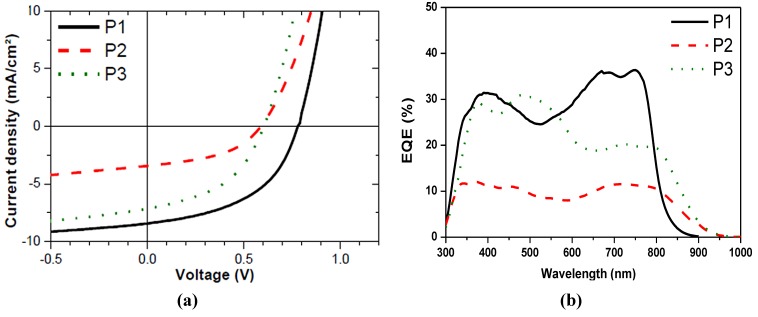
(**a**) IV-characteristics; and (**b**) EQE of devices based on polymer: PC_71_BM-based blends.

Blends with the same weight ratio of polymer and PC_71_BM, solvent and amount of additive have been used to prepare photovoltaic devices. The best devices of concentration, thickness, and 1,8-diiodooctane (DIO) addition/exclusion variation are presented (Figure 2a) . In this study, devices based on P1 and P3:PC_71_BM blends produce similar values for J_sc_ and FF while a drastically lowered voltage limits the performance of P2 (Table 3). The voltage difference corresponds for P2 and P3 to the observed difference in energy gap due to the alkoxy substitution. When comparing P1 and P3, the somewhat lower J_sc_, which is mainly reflected in the lower external quantum efficiency (EQE) in the 600–800 nm absorption region (Figure 2b), could originate from a more unfavorable blend morphology that hampers efficient free charge generation or charge extraction from excitons generated on the polymer. When comparing the performance of P2 and P3, the V_oc_ and FF are rather similar but the photovoltaic performance of P2 is even further reduced due to a low current.

**Table 3:** Photovoltaic data of devices based on polymer: PC_71_BM blends.

Material	Polymer:PC_71_BM (w:w)	Thickness (nm)	RMS blend (nm)	J_sc_ (Ma/cm^2^)	V_oc_ (mV)	FF	η (%)
P1	1:2	125	2.77	8.4	780	49	3.2
P2	1:2	80	10.9	3.4	590	45	0.9
P3	1:2	77	4.27	7.1	600	46	2.0

Active layers spun from 5 to 15 mg/mL polymer: CHCl_3_ with 23 mg/mL DIO. Device architecture ITO/PEDOT: PSS/active layer/Ca/Ag.

**Figure 3:**
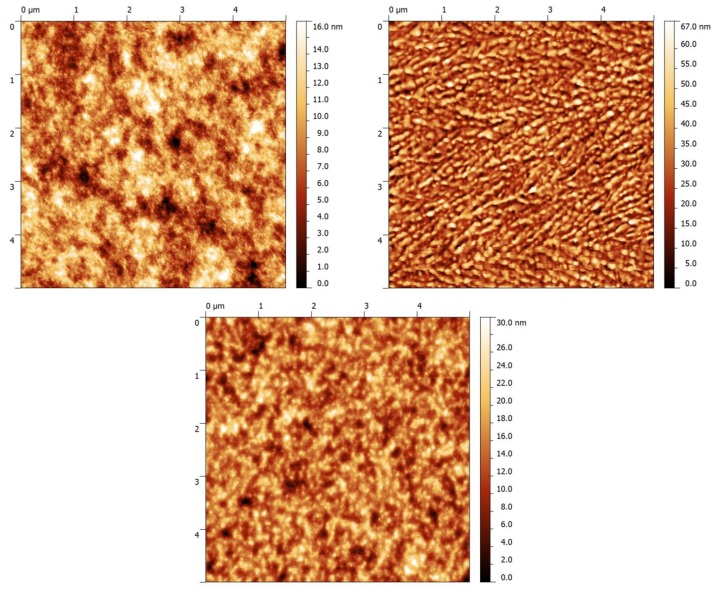
AFM topographical images (5 μm × 5 μm) for the P1, P2, and P3:PC_71_BM blends respectively.

AFM imaging (Figure 3) reveals similar surface roughness and blend morphology for both the P1 and P3:PC_71_BM-based blends. In contrast, the P2:PC_71_BM blend shows increased surface roughness and a morphology that vastly deviates from the other two blends. The observed difference could be responsible for the reduced current density compared to the other two polymers: fullerene blends.

## 3. Experimental Section

### 3.1. Experimental Details

Unless otherwise stated, all reactions were done under nitrogen. Tetrahydrofuran (THF) was dried on sodium + benzophenon and distilled prior to use. The 3,6-bis(5-bromothiophen-2-yl)-2,5-bis(2-hexyldecyl)pyrrolo[3,4-c]pyrrole-1,4(2H,5H)-dione monomer was kindly provided by BASF. 1,4-bis(4,4,5,5-tetramethyl-1,3,2-dioxaborolan-2-yl)benzene was bought from Sigma Aldrich (MO, USA) and recrystallized twice from ethanol prior to use, while 2,2′-(2,5-dimethoxy-1,4-phenylene)bis(4,4,5,5-tetramethyl-1,3,2-dioxaborolane) and 2,2′-(2,5-bis(octyloxy)-1,4-phenylene)bis(4,4,5,5-tetramethyl-1,3,2-dioxaborolane) [34], 3-(5-bromothiophen-2-yl)-2,5-bis(2-hexyldecyl)-6-(thiophen-2-yl)pyrrolo[3,4-c]pyrrole-1,4(2H,5H)-dione oligomers [35], and polymers [19] were synthesized according to slightly modified literature procedures. All other chemicals and solvents were bought from Sigma-Aldrich and used as received. Synthetic details are included in the supporting information.

### 3.2. Characterization

1H-NMR and 13C NMR spectra have been measured on a Varian 400/54/ASP with CDCl3 as the solvent. In all cases, the peak values were calibrated relative to tetramethylsilane. Size exclusion chromatography (SEC) was performed on Waters Alliance GPCV2000 with a refractive index detector columns: Waters Styvagel HT GE×1, Waters Styvagel HMW GE×2. The eluent was 1,2,4-trichlorobenzene. The operating temperature was 135 °C, and the dissolution time was 2 h. The concentration of the samples was 0.5 mg/mL, which were filtered (filter: 0.45 µm) prior to analysis. The molecular weights were calculated according relative calibration with polystyrene standards. UV-Vis/near IR absorption spectra were measured with a Perkin Elmer Lambda 900 UV-Vis-NIR absorption spectrometer. For the solution absorption measurements, chloroform solutions of polymer with concentrations ranging from 0.0155 to 0.0175 g/L have been prepared. The mass absorption coefficient was then calculated, and corrected for the dilution effect of the alkyl side chains by multiplying with a correction factor based on the repeating unit mass. Abs_corr_ = Abs * (mass repeating unit_P2 or P3_/mass repeating unit_P1_).TGA measurements were done on a Perkin Elmer TGA7 Thermo Graphic Analyzer, temperature range 30–600 °C, heating rate 10 °C/min. DSC measurements were done on a Perkin Elmer Pyris, temperature range 30–300 °C, heating/cooling rate 10 °C/min, second scan used after baseline subtraction. Square-wave voltammetry (SWV) measurements were carried out on a CH-Instruments 650A Electrochemical Workstation. A three-electrode setup was used with platinum wires both as working electrode and counter electrode, and Ag/Ag^+^ used as reference electrode calibrated with Fc/Fc^+^. A 0.1 M solution of tetrabutylammoniumhexafluorophosphate (Bu4NPF6) in anhydrous acetonitrile was used as supporting electrolyte. The polymers were deposited onto the working electrode from chloroform solution. In order to remove oxygen from the electrolyte, the system was bubbled with nitrogen prior to each experiment. The nitrogen inlet was then moved to above the liquid surface and left there during the scans. HOMO and LUMO levels were estimated from peak potentials of the third scan by setting the oxidative peak potential of Fc/Fc^+^ *vs.* the normal hydrogen electrode (NHE) to 0.630 V [36], and the NHE *vs.* the vacuum level to 4.5 V [37].

Specular X-ray diffraction (sXRD) measurements were recorded at room temperature on a Bruker D8 Advance diffractometer using Cu-K_α_ radiation (λ = 1.5418 Å) and equipped with an MRI (Material Research Instruments) heating stage for temperature-dependent measurements. The angular resolution was 0.02° (0.006°) per step with a typical counting time of 10 s for sXRD. For sXRD measurements (powder samples), a few mg of the polymer was deposited on an aluminiumplate (10 × 20 × 0.5 mm³), as it conducts heat. However, this substrate gives additional reflections at *ca.* 24.1° and 38.5° in 2θ.

Solar cell fabrication and characterization indium-tin-oxide (ITO) coated glass substrates, purchased from Kintec with 10 Ω/sq, were cleaned in a sequence of detergent, deionized water, acetone, and isopropanol, each step for 10 min in an ultrasonic bath. The cleaned substrates were further purified by UV-ozone treatment for 15 min. The substrates were then spin coated with a 0.45 µm filtered poly(3,4-ethylenedioxythiophene):poly(styrenesulfonate) (PEDOT:PSS) solution, Clevios PH500 purchased from HC Starck, at 5000 rpm for 60 s to produce a 25 nm thick film. Afterwards, the substrates were transferred to a nitrogen glovebox where the further steps of the sample fabrication process were performed. The substrates were subsequently heated on a hotplate at 130 °C for 10 min to remove residual water. The active layers containing, as acceptor, PC_71_BM (98% nano-c) were prepared with concentrations of 5 mg/mL to 15 mg/mL, dissolved in chloroform (99% Merck) with 23 mg/mL 1,8-diiodooctane (98% Sigma-Aldrich), and stirred for at least 15 h. DCB as a solvent did not result in solutions for P1 and P2. The films were prepared by spin coating at spin speeds of 600 rpm to 1000 rpm. The top electrodes of 20 nm calcium/150 nm silver were deposited in a high vacuum chamber at pressures of p < 5 × 10^−7^ torr on cooled substrates with temperatures of T < 0 °C. The photovoltaic characteristics were measured under nitrogen atmosphere using a Keithley 2602A source meter under 100 mW/cm^2^ AM1.5 simulated illumination using an Abet Technologies Sun 2000 solar simulator with a 550 W Xenon arc lamp. The active areas of the devices of about 0.03 cm^2^ were individually determined under an optical microscope.

Theoretical calculations were done with Gaussian09 [38], DFT using hybrid functional B3LYP [39,40,41] with a valence triple-zeta Gaussian basis set, plus d-functions on heavy elements and p-functions on hydrogen, 6-31G (d,p) were used throughout the study. All geometries were optimized and local minima were checked with second derivatives.

## 4. Conclusions

Besides influencing energy levels, side chain modifications on conjugated polymers significantly alter the physical behavior of polymers, which affects the blend morphology obtained from solution processing and thus photovoltaic performance. These results show that supposed small structural alterations such as methoxy substitution already significantly alter the physical properties of the parent polymer and also that oligomers and polymers respond divergent to structural alterations made on a parent structure due to conformational distribution that can arise in the polymer chain.

## Supplementary Materials

Supplementary materials can be accessed at: http://www.mdpi.com/1996-1944/6/7/3022/s1.
