# Supplementary material for: The Influence of Alkoxy Substitutions on the Properties of Diketopyrrolopyrrole-Phenyl Copolymers for Solar Cells
Source: Materials (Basel). 2013 Jul 22;6(7):3022–34. doi: 10.3390/ma6073022 (PMC5521293; doi:10.3390/ma6073022)
Supplement: Supplementary file 1 [file materials-06-03022-s001.pdf]

Materials **2013**, *6*, 3022-3034; doi:10.3390/ma6073022

OPEN ACCESS

materials

ISSN 1996-1944

www.mdpi.com/journal/materials

Article

Supporting Information

Synthetic Procedures

Oligomer Synthesis

3-(5-bromothiophen-2-yl)-2,5-bis(2-ethylhexyl)-6-(thiophen-2-yl)pyrrolo[3,4-c]pyrrole-1,4(2H,5H)-dione (0.048 mmol), the phenyl or dimethoxypinacoldiboronic esters (0.024 mmol), tri(dibenzylidene-acetone)palladium(0)($Pd_2(dba)_3$) (2.5 µmol), tri-o-tolyl phosphine (10 µmol) and potassium carbonate (0.24 mmol) were dissolved in a 9:1 THF:water mixture (30 min degassed with N_2) and reacted while stirring at 70 ℃ while monitoring the reaction by TLC (40:60 $CHCl_3$:hexanes). After 1h the reaction was complete, judged by disappearance of the starting materials and the organic products extracted into CHCl3. Two other main spots besides the product material were observed. Analysis by MALDI-TOF revealed that the masses corresponded to the homocoupled and debrominated DPP reactant. After evaporation and extensive purification by either chromatography or preparative TLC (40:60 $CHCl_3$:hexanes) the oligomers were obtained as dark blue, somewhat tacky solids.

6,6'-(5,5'-(1,4-phenylene)bis(thiophene-5,2-diyl))bis(2,5-bis(2-hexyldecyl)-3-(thiophen-2-yl)pyrrolo[3,4-c]pyrrole-1,4(2H,5H)-dione) (O1)

Yield after purification, 8 mg (11%) (^{1}H NMR (400 MHz, Chloroform-d) δ 8.95 (d, *J* = 4.1 Hz, 2H), 8.89 (d, *J* = 3.9 Hz, 2H), 7.72 (s, 4H), 7.62 (d, *J* = 4.4 Hz, 2H), 7.52 (d, *J* = 4.1 Hz, 2H), 7.28 (dd, *J* = 5.0 Hz, 3.9 Hz, 2H), 4.05 (dd, *J* = 12.4, 7.7 Hz, 8H), 2.05–1.84 (dm, 4H), 1.42–1.10 (m, 96H), 0.90–0.77 (m, 24H). MALDI (m/z) calculated (M^+): 1571.02, found: 1570.717.

6,6'-(5,5'-(2,5-dimethoxy-1,4-phenylene)bis(thiophene-5,2-diyl))bis(2,5-bis(2-hexyldecyl)-3-(thiophen-2-yl)pyrrolo[3,4-c]pyrrole-1,4(2H,5H)-dione) (O2)

Yield after purification: 10 mg (13%) (^{1}H NMR (400 MHz, Chloroform-d) δ 9.07 (d, *J* = 4.3 Hz, 2H), 8.87 (d, *J* = 3.8 Hz, 2H), 7.70 (d, *J* = 4.4 Hz, 2H), 7.61 (d, *J* = 4.9 Hz, 2H), 7.37 (s, 2H), 7.26 (dd, *J* = 5.0, 4.0 Hz, 2H), 4.10 (d, *J* = 7.6 Hz, 4H), 4.04 (d, *J* = 7.8 Hz, 4H), 4.04 (2, 6H) MeO overlaps with N–CH_2, 2.09–1.97 (m, 2H), 1.97–1.85 (m, 2H), 1.41–1.12 (m, 96H), 0.91–0.76 (m, 24 H). MALDI (m/z) calculated (M^+): 1631.04, found: 1631.03.

Typical Polymer Workup and Characterization

The polymerization reaction mixture were precipitated in MeOH and collected via filtration. Thepolymer was then redissolved in chloroform and vigorously stirred for 1 h with a 10% water solution

of sodium diethyldithiocarbamatetrihydrate at 60 ℃. The organic and aqueous layers are separated and the organic layer washed 3 times with demiwater. The organic layer is concentrated to about 20 mL and precipitated in methanol. The resulting dark blue fibers are then collected by filtration, transferred to a Soxhlet thimble and extracted subsequently with methanol, diethyl ether, acetone, hexanes, ethyl acetate and finally chloroform. The chloroform solution is again reduced to about 20 mL via rotary evaporation and precipitated in methanol, resulting in dark blue fibers. Collection by vacuum filtration and drying overnight under vacuum at 40 ℃ yielded the polymer as dark colored solids.

1. P1 was obtained as dark purple fibers (96%), ^{1}H-NMR (400 MHz, $CDCl_3$): δ (ppm), 9.4–8.6 (b, ArH), 8–7.2 (b, ArH), 7.2–6.4 (b, ArH), 4.5–3 (b, N–CH_2), 2–1 (b, alkyl), 1–0.7 (b, CH_3).
2. P2 was obtained as dark blue fibers (94%), ^{1}H-NMR (400 MHz, $CDCl_3$): δ (ppm), 9.4–8.6 (b, ArH), 8–7.2 (b, ArH), 7.2–6.4 (b, ArH), 4.4–3.5 (b, N–CH_2), 2.2–1.8 (b, O–CH_3), 1.8–1 (b, alkyl), 1–0.7 (b, CH_3).
3. P3 was obtained as dark blue fibers (77%), ^{1}H-NMR (400 MHz, $CDCl_3$): δ (ppm), 9.4–8.6 (b, ArH), 8–7.2 (b, ArH), 7.2–6.4 (b, ArH), 4.5–3.6 (b, N–CH_2), 2.2–1.8 (b, O-alkyl), 2–1 (b, alkyl), 1–0.6 (b, CH_3).

DSC and TGA Measurements on Polymers

Figure S1. (**a**) Thermal stability of all polymers under N_2; (**b**) DSC thermogram of P1; (**c**) DSC thermogram of P2, the high temperature region shows a dip which was attributed to thermal degradation of the material; and (**d**) DSC thermogram of P3.

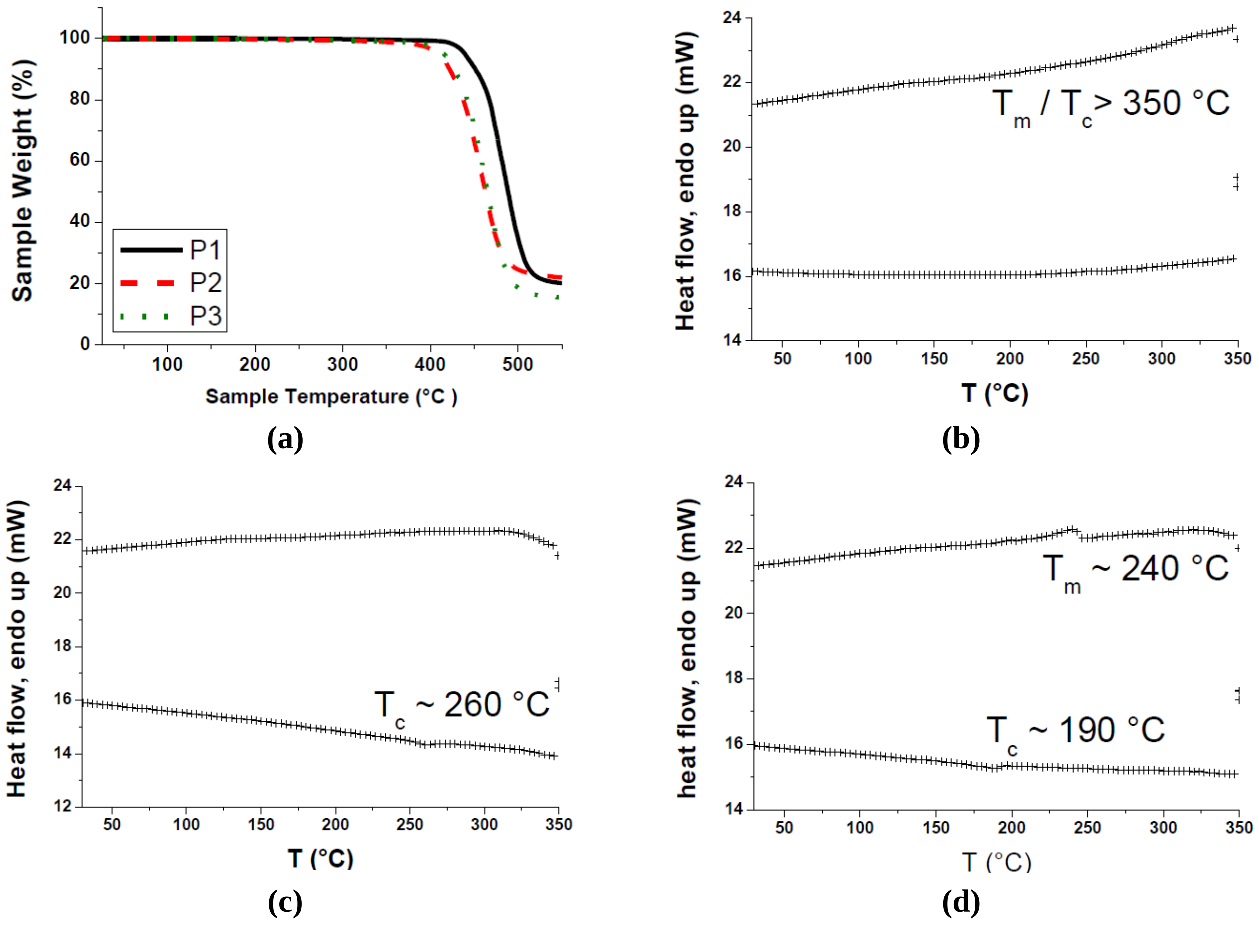

DSC and TGA Measurements on OLigomers

Figure S2. (**a**) DSC; and (**b**) TGA thermograms of the oligomers.

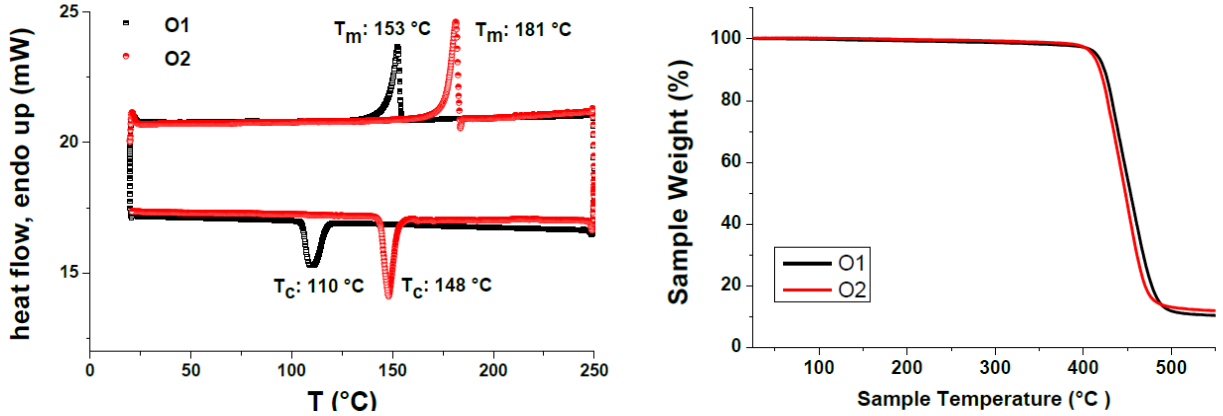

(a) **(b)**

Results X-Ray Diffraction

Figure S3. XRD spectra of the polymers.

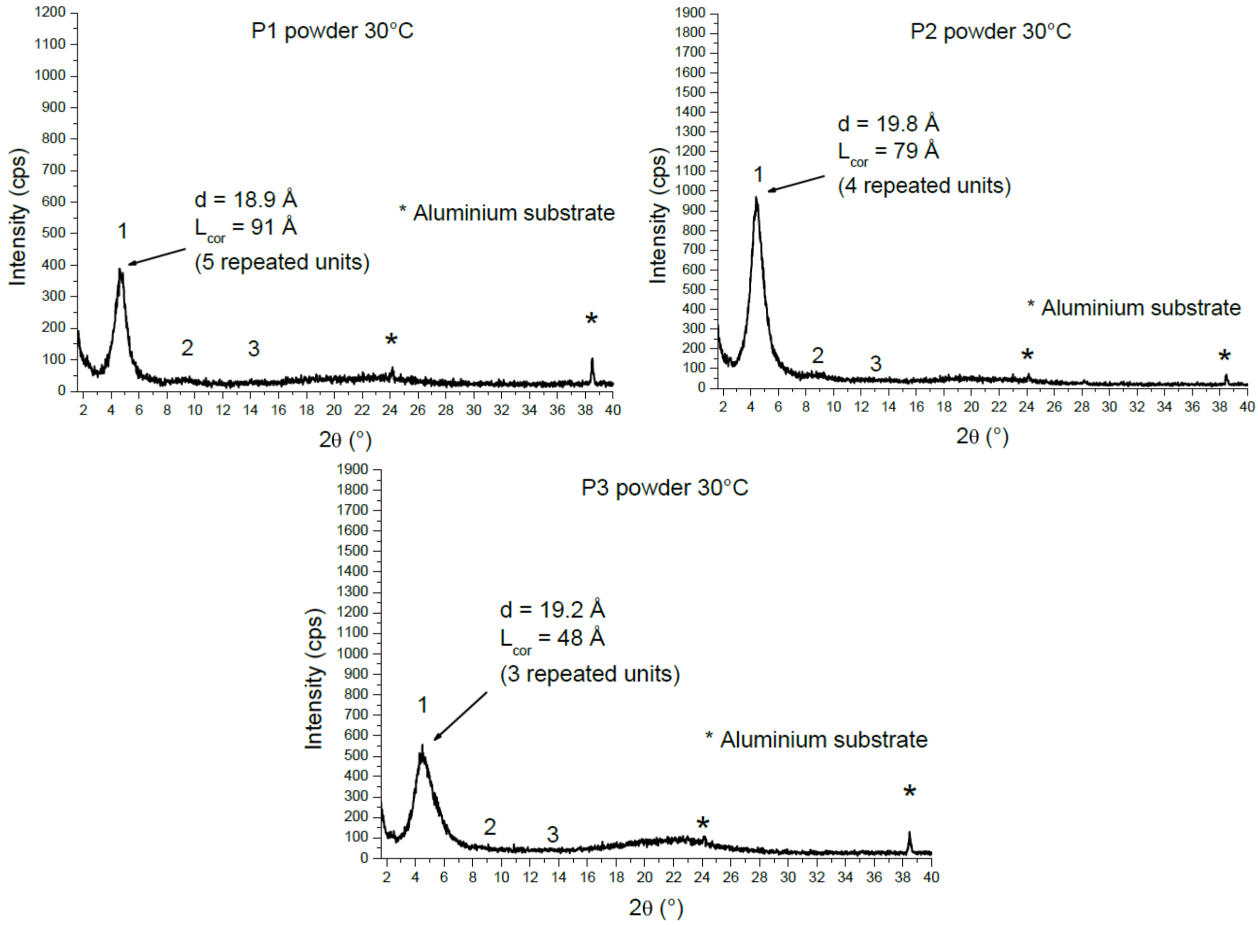

Figure S4. SWV of P1, P2, P3 and $PC_{70}BM$. Electrochemical studies.

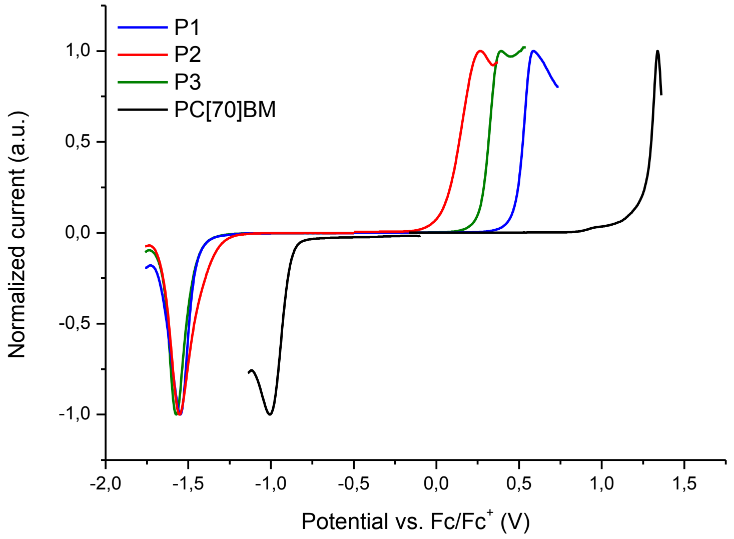

Figure S5. UV-Vis spectra of oligomers in solution (chloroform) and solid state (dropcastedfromchloroform). Oligomer solution and thin film absorption.

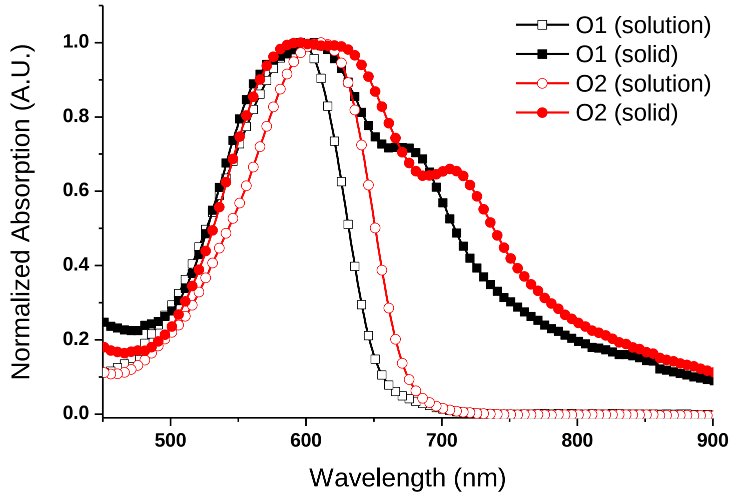

Theoretical Calculations

Scheme S1. Calculated bond lengths (R) and dihedral angles (D) of O1 and O2.

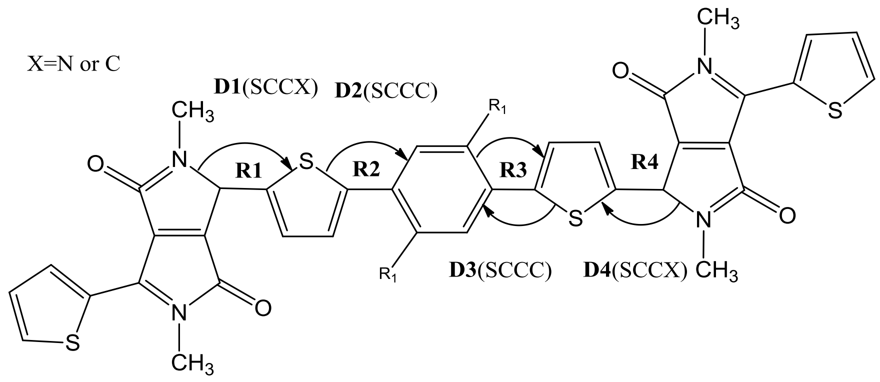

Table S1. Bond length and dihedral angle after geometrical optimization with B3LYP/6-31G(d,p).

Molecule	Bond length R (Å)					Dihedral angle D (°)			
	R_1	R1	R2	R3	R4	D1(SCCX)	D2(SCCC)	D3(SCCC)	D4(SCCX)
O1a	H	1.437	1.460	1.461	1.437	0.0	−20.7	−20.7	0.7
O1b	H	1.437	1.460	1.461	1.437	1.5	−20.8	−21.0	1.2
O1c	H	1.437	1.461	1.462	1.438	0.5	−20.8	−20.4	11.1
O1d	H	1.439	1.462	1.461	1.437	10.2	−21.7	−22.0	0.2
O2a	OCH_3	1.436	1.461	1.461	1.436	−1.5	−15.2	−15.2	−2.1
O2b	OCH_3	1.436	1.460	1.461	1.436	0.5	20.5	−12.8	−1.0
O2c	OCH_3	1.436	1.460	1.461	1.436	0.2	−20.4	−20.6	0.8
O2d	OCH_3	1.438	1.461	1.461	1.435	8.8	−7.8	11.6	1.0
O2e	OCH_3	1.438	1.462	1.461	1.436	−10.6	22.8	−10.6	−0.9

Scheme S1. Energetic scheme of different conformers of (**a**) O1; and (**b**) O2 and some Transition States (TS) as optimized with B3LYP/6-31G(d,p). All energies in eV.

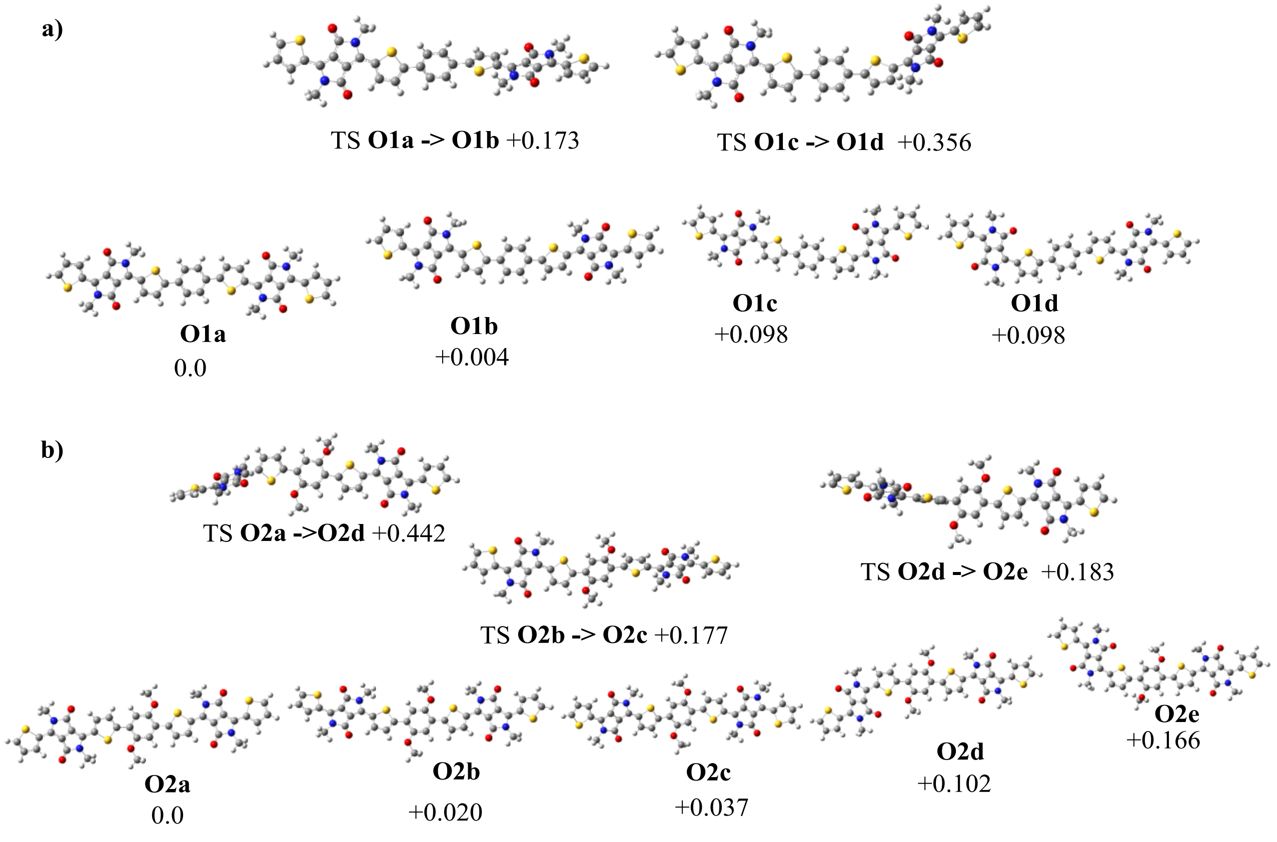

Different conformers of oligomers O1 and O2 were optimized with B3LYP/6-31G(d,p) and some geometrical parameters, bond lengths and dihedral angles, are shown in Table S1. For computational reasons the side chain on the DPP unit are shortened to a methyl group. Most dihedral angles and the total pitch are larger for O1 compared with O2 conformers but bond lengths are essentially the same. Rotational barriers are in the range of 0.17–0.44 eV and seems to be slightly larger for the dimethoxy substituted oligomer which would decrease the entropy and thus result in higher T_m and T_c.

Polymer Solutions

Figure S6. (**a**) UV-Vis absorption of hot and RT polymer:CN solutions; and (**b**) recovery speed of RT UV-Vis absorption from hot UV-Vis absorption.

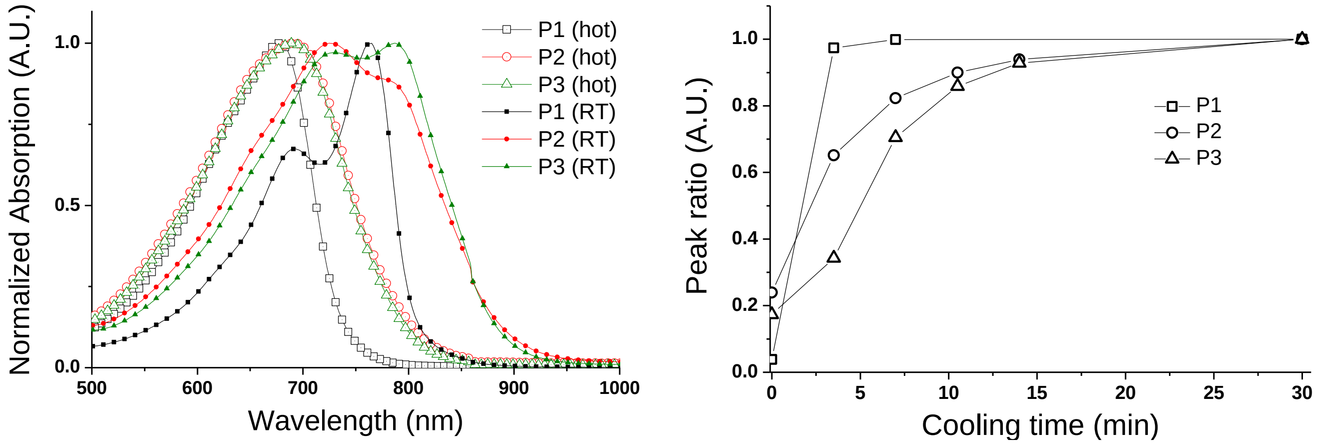

(a) **(b)**

Starting from a hot polymer: 1-chloronaphthalene solution (~16 mg/mL) with starting temperature ~100 ℃, time interval corresponds to the time needed for 1 scan from 1200 to 300 nm. Temperature intervals roughly correspond to 100–60–42–32–28 ℃. Ratio is calculated between the redshifted low energy peak at RT and at elevated temperatures, as function of cooling time. The closer the ratio becomes 1, the more the spectrum at time t resembles the spectrum at RT. Lines function as guide to the eye.

Figure S7. Polymers in different solvents and at different temperatures. In all cases, left vial is P1, middle vial is P2 and right vial is P3. (**a**) Chloroform solutions; (**b**) hot 1-chloronaphthalene solutions; (**c**) 1-chloronaphthalene solutions at RT; and (**d**) 1-chloronaphthalene solutions after 16 h. Notice the P1 polymer being precipitated, which does not occur for chloroform solutions.

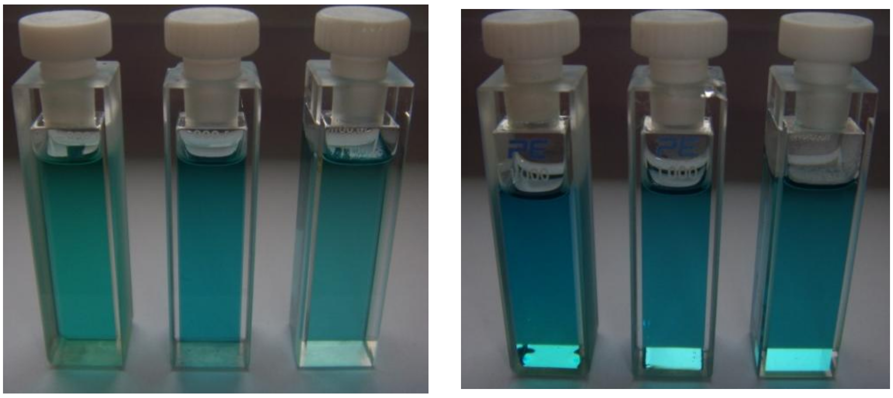

(a) **(b)**

Figure S7. *Cont.*

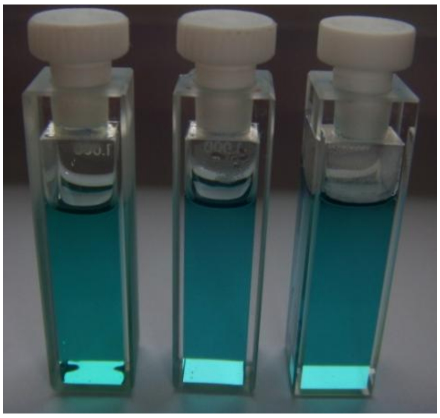

(c)

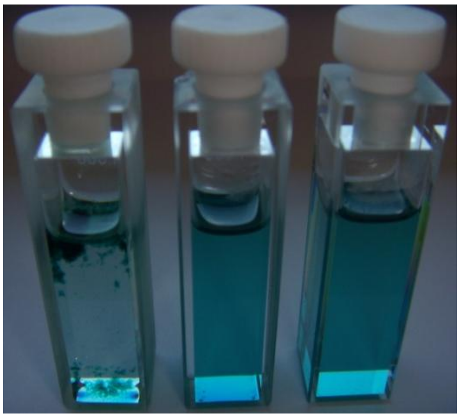

(d)

© 2013 by the authors; licensee MDPI, Basel, Switzerland. This article is an open access article distributed under the terms and conditions of the Creative Commons Attribution license (http://creativecommons.org/licenses/by/3.0/).
